# Frequency of Traumatic Ulcerations and Post-insertion Adjustment Recall Visits in Complete Denture Patients in an Iranian Faculty of Dentistry

**DOI:** 10.5681/joddd.2011.010

**Published:** 2011-06-14

**Authors:** Katayoun Sadr, Farhang Mahboob, Elaheh Rikhtegar

**Affiliations:** ^1^ Assistant Professor, Department of Prosthodontics, Faculty of Dentistry, Tabriz University of Medical Sciences, Tabriz, Iran; ^2^ Dental and Periodontal Research Center, Faculty of Dentistry, Tabriz University of Medical Sciences, Tabriz, Iran; ^3^ Dentist, Private Practice, Tabriz, Iran

**Keywords:** Post-insertion care, removable complete denture, traumatic mucosal ulceration

## Abstract

**Background and aims:**

The first few days following the insertion of complete dentures are critical for the patients since they are struggling to adapt to their new dentures. This study aimed to evaluate the most common locations of traumatic ulcerations, their frequency and also the duration and number of adjustment visits required to achieve patient comfort fol-lowing placement of complete dentures.

**Materials and methods:**

Sixty patients were selected from edentulous patients referring to a removable prosthodontics department. Complete dentures were fabricated for the patients. After placement of dentures, all the patients were evaluated from the day after placement until patient comfort was achieved. Descriptive analysis was performed and chi-squared test was used to evaluate the associations between lesions, post-insertion visits and gender.

**Results:**

A total of 85.8% of patients required denture adjustment because of mucosal injuries during their first visit fol-lowing denture placement. Maxillary and mandibular dentures did not require further adjustments after fourth and sixth visits, respectively. No significant differences were detected between males and females in the number of mucosal injuries in the anatomical areas evaluated in the maxilla and mandible using Fisher's exact test (P > 0.05). Furthermore, the number of mandibular dentures requiring adjustments was significantly higher than maxillary dentures in all the post-insertion ap-pointments (P < 0.001).

**Conclusion:**

Since most mucosal injuries are located in the vestibule, adequate extension of denture flanges, especially during border molding, and use of pressure indicators that reveal over-extended borders, play an important role in decreas-ing mucosal injuries and improving patient comfort following complete denture placement.

## Introduction

Placement of a complete denture is not the final step in the treatment of edentulous patients and patient's visit to the dentist continues long after that.^1^ Traumatic mucosal ulcerations are among the most common complaints of edentulous patients after placement of complete dentures. ^2,3^ Ulcers are painful and result in patient discomfort. As a result, patients are no longer willing to wear their dentures and develop a feeling of mistrust toward their dentist and his/her treatment plan.^2^ Various factors are responsible for mucosal injuries among which denture base defects, like overextension of the flanges, improper adaptation of internal surface of the denture with underlying soft tissues, denture irregularities, porosities or tissue undercuts and presence of immature occlusal contact are the most common.^1^ The above-mentioned defects are usually due to clinical or laboratory errors during various stages of denture fabrication, including border molding, impression taking, and inadequate or insufficient polishing of the denture.^2-8^

Brunello and Mandikos^3^ showed in 100 patients with newly fabricated complete dentures that the most common post-insertion complaint was pain and discomfort due to mucosal injuries and traumatic ulcerations, which had no significant association with patient age, sex or medical status. However, significant relationships were observed between denture design faults, or the condition of the patient’s mucosa and patient complaints. Drago^5^ did not report any significant differences in the number of adjustment visits required for patients whose dentures were fabricated with the use of different impression techniques or border molding methods. He also reported that appropriate condition of clinical and laboratory phases play a major role in decreasing post-insertion problems for patients. Dervis^6^ in his study assessed the most common complaints of 600 patients three months after insertion of their new dentures. However, no significant relationship was found when age, gender, and medical status were compared with patient complaints. However, statistically significant relationships were observed between denture construction faults or the condition of the patient’s denture-bearing mucosa and patient complaints. Laurina and Soboleva^7^ conducted a retrospective study with information derived from literature from 1984 to 2004 on patients who experienced ongoing difficulties with new complete dentures to determine possible underlying causes. They reported that in most instances, complete denture patients present with complaints only when there is real denture construction or design fault. Kivovics et al^2^ evaluated mucosal tissues of 61 edentulous healthy patients who were wearing newly fabricated complete dentures. They reported that it is not always easy to record mucogingival junction by various impression techniques and there is always a risk of overextension of flanges towards the mobile mucosa, which irritates the tissue and results in mucosal injuries in those areas. Furthermore, many clinicians try to extend the flanges and denture bases as much as possible in order to achieve better retention, especially in the mandible, which results in overextension of the flanges towards the mobile mucosa.

As noted in the above-mentioned studies, most patient complaints in the first few days after insertion of the complete denture are due to a fault or failure in one or more phases of denture construction process. Therefore, if there is some information regarding the most frequent locations of mucosal injuries and traumatic ulcers after placement of complete dentures, we can thoroughly revise and refine our denture reconstruction method. By making some small changes or applying simple strategies during denture fabrication or before its insertion, we may be able to prevent mucosal injuries and decrease post-insertion problems. This study aimed at determining the frequency and common locations of mucosal ulcerations and the duration of adjustment visits required to achieve patient comfort.

## Materials and Methods

In this descriptive analytical study, 60 edentulous patients (23 females and 37 males) who were referred to the Removable Prosthodontics Department at Faculty of Dentistry, Tabriz University of Medical Sciences, Iran, in 2009, were selected after reviewing medical and dental histories and examining their oral tissues. The mean age of the patients was 51.01 ± 9.24 years (a range of 27–70 years). The inclusion criteria were:

1. No previous history of diabetes, immunologic diseases, neurologic diseases, mental disorder, xerostomia and bruxism
2. Complete healing of the socket and the extraction site in those who were getting their first complete dentures
3. No hyperplastic or inflammatory mucosal lesions
4. No Candida infections
5. No history of autoimmune diseases associated with recurrent mucosal ulcers
6. No bony undercuts or irregularities in denture’s insertion path
7. No tobacco use
8. No pregnancy and no use of contraceptives in women
9. Age younger than 70 years

Complete dentures were fabricated for all the patients by using the same method and materials, advocated by Zarb and Bolender.^1^ All the phases were controlled and supervised by a prosthodontist. The patients were visited on the first, second and third days after insertion and then every two days until the resolution of all the mucosal injuries. At each appointment, the location of the lesion and the part of denture requiring adjustment were marked and recorded. Descriptive statistical analysis was performed using SPSS ver.13 software. Chi-squared test was used to evaluate the correlation between mucosal injuries and post-insertion day and the relationship between lesions and patient gender. Statistical significance was defined at P < 0.05.

## Results

The patients were visited on the first, second and third days after insertion and then every two days until the resolution of all the mucosal injuries. As seen in Table 1, in the first visit 43 out of 60 maxillary dentures and all the 60 mandibular dentures needed adjustments because they had caused mucosal injuries and patient discomfort. During the following appointments, the need for adjustments decreased gradually. In the fourth visit, none of the maxillary dentures required adjustment. However, mandibular dentures required adjustments untill the sixth visit (Table 1). Chi-squared test showed a significant correlation between the number of maxillary (λ2=155.221, df=6, P=0.001) and mandibular (λ2=258.995, df=6, P=0.001) dentures requiring adjustments and post-insertion visits.

**Table 1:** Number of patients requiring maxillary and mandibular dentures adjustments after denture placement

	Total	Adjustment 1	Adjustment 2	Adjustment 3	Adjustment 4	Adjustment 5	Adjustment 6	Adjustment 7
Maxilla	60	43 71.7%	32 53.3%	23 38.3%	13 21.7%	0	0	0
Mandible	60	60 100%	58 96.7%	54 90%	46 76.7%	22 36.7%	8 13.3%	0
Total	120	103 85.8%	90 75%	77 64.1%	59 49.1%	22 18.3%	8 6.6%	0

The results showed that the number of mandibular dentures requiring adjustments was significantly higher than maxillary dentures in all the post-insertion appointments (λ2=78.838, df=1, P=0.001).

Most frequently injured maxillary areas were posterior palatal seal area in the soft palate (27%), buccal slope of the residual ridge (13.8%), distobuccal sulcus (13.1%) and labial frenum (9.9%). In the mandible, the most frequently injured areas were retromylohyoid area (48.6%), buccal sulcus adjacent to the buccal shelf (9.8%), retromolar pad (9.5%) and frenum (8.1%) (Tables 2 and 3).

The least common locations for maxillary ulcerationss were hard palate and mid-palatal suture (0%), incisive papilla and rugae (0.65%), tuberosity (2.6%), and buccal and labial sulci (4.6%). The lowest frequency of lesions in the mandible was seen in the sublingual fold (0%), labial sulcus and mylohyoid region of the lingual sulcus (1.2%) and buccal frenum and buccal shelf (2.1%) (Tables 2 and 3).

No significant differences were detected between males and females in terms of mucosal injuries in the above-mentioned anatomic areas of the maxilla and mandible using Fisher’s exact test (P > 0.05).

## Discussion

Patients usually need special attention on behalf of their dentists during the first few days after insertion of their complete dentures. In the present study approximately 85.8% of patients required adjustments in the first 24 hours after insertion. This rate was 75% in the second appointment, 64.15% in the third, 49.15% in the fourth, 18.3% in the fifth, and 6.6% in the sixth appointment. These rates indicate that patient care does not end by insertion of complete dentures. The results of the present study are consistent with those of Kivoviks et al,^2^according to whom 87% of patients in his study required adjustments in their first post-insertion appointment.^2^ The above-mentioned rates also show the healing process of ulcerations since after six visits during 2 week after insertion, no further adjustments were required for dentures.

All the patients (100%) required mandibular denture adjustments in their first post-insertion appointment. The number of mandibular mucosal injuries was also significantly higher than those of maxilla in all the post-insertion appointments and number of adjustment visits was higher for mandibular dentures. These results were not unexpected but indicate that more attention should be paid to border molding, final impression taking, and adaptation of denture and proper extension of flanges when fabricating mandibular dentures. Mandibular denture has less support and retention compared to maxillary denture due to a smaller denture-bearing area.^1^ Presence of the tongue and greater movement range of mandibular denture make this situation more complicated.^1^

**Table 2:** Number of maxillary injuries related to clinical anatomic sites and gender (60 patients and 152 corrections)

Anatomical sites	Adjustment 1			Adjustment 2			Adjustment 3			Adjustment 4			Adjustment 5			Total
	M	F	Total	M	F	Total	M	F	Total	M	F	Total	M	F	Total	
Labial frenum	7	4	11	1	1	2	0	1	1	0	1	1	0	0	0	15
Buccal frenum	4	2	6	2	0	2	1	1	2	1	0	1	0	0	0	11
Maxillary tuberosity	1	0	1	1	1	2	1	0	1	0	0	0	0	0	0	4
Hamular notch	0	4	4	1	3	4	1	2	3	1	1	2	0	0	0	13
PPS	8	1 0	18	5	8	13	3	3	6	2	2	4	0	0	0	41
Incisive papilla	1	0	1	0	0	0	0	0	0	0	0	0	0	0	0	1
Midpalatal suture	0	0	0	0	0	0	0	0	0	0	0	0	0	0	0	0
Rugae	0	0	0	0	0	0	0	1	1	0	0	0	0	0	0	1
Palatal torus	0	1	1	0	0	0	0	0	0	0	0	0	0	0	0	1
Hard palate	0	0	0	0	0	0	0	0	0	0	0	0	0	0	0	0
Crest of residual ridge	3	2	5	2	1	3	1	0	1	1	0	1	0	0	0	10
Labial sulcus	2	1	3	3	1	4	0	0	0	0	0	0	0	0	0	7
Buccal sulcus	1	0	1	1	0	1	2	1	3	2	0	2	0	0	0	7
Distobuccal sulcus	2	3	5	5	3	8	2	2	4	2	1	3	0	0	0	20

**Table 3:** Number of mandibular injuries related to clinical anatomic sites and gender (60 patients and 368 corrections)

Anatomical sites	Adjustment 1			Adjustment 2			Adjustment 3			Adjustment 4			Adjustment 5			Adjustment 6			Adjustment 7				Total
	M	F	Total	M	F	Total	M	F	Total	M	F	Total	M	F	Total	M	F	Total		M	F	Total	
Labial frenum	8	4	12	7	2	9	4	1	5	2	1	3	1	0	1	0	0	0		1	0	0	30
Buccal frenum	2	1	3	1	0	1	1	1	2	1	1	2	0	0	0	0	0	0		0	0	0	8
Retromolar pad	6	5	11	8	5	13	3	4	7	1	2	3	0	1	1	0	0	0		0	0	0	35
Plica sublingualis	0	0	0	0	0	0	0	0	0	0	0	0	0	0	0	0	0	0		0	0	0	0
Mylohyoid ridge	3	0	3	2	1	3	2	1	3	2	2	4	1	1	2	0	0	0		1	0	0	15
Mandibular torus	0	0	0	0	0	0	0	0	0	0	0	0	0	0	0	0	0	0		0	0	0	0
Lingual sulcus (premylohyoid)	2	0	2	1	1	2	0	2	2	1	3	4	1	0	1	1	0	1		1	0	0	12
Lingual sulcus (mylohyoid)	1	0	1	0	1	1	1	0	1	1	0	1	1	0	1	0	0	0		1	0	0	5
Lingual sulcus (retromylohyoid)	29	17	49	27	17	44	25	15	40	17	13	30	7	6	13	5	1	6		7	0	0	179
Slope of residual ridge	2	3	5	2	3	5	1	5	6	0	2	2	1	0	1	1	0	1		1	0	0	20
Crest of residual ridge	7	0	7	4	0	4	1	1	2	1	2	3	0	0	0	0	0	0		0	0	0	16
Labial sulcus	0	1	1	1	0	1	0	1	1	0	1	1	0	0	0	0	0	0		0	0	0	4
Buccal sulcus (buccal shelf)	7	3	10	6	4	10	4	4	8	4	2	6	2	0	2	0	0	0		2	0	0	36
Distobuccal sulcus	0	1	0	1	0	1	0	3	3	0	1	1	0	2	2	0	0	0		0	0	0	8
Total	67	35	102	60	34	94	42	38	80	30	30	60	14	10	24	7	1	8		14	0	0	368

According to Kivoviks et al^2^ dentists tend to extend flanges as much as possible to overcome the retention problem and that is why the highest frequency of injuries were seen in borders and flanges in the retromylohyoid area (48.6%), the buccal sulcus adjacent to the buccal shelf (9.8%), and the retromolar pad (9.5%). By applying a pressure indicating paste and detection of overextended borders/flanges at delivery or post-insertion stages, mucosal injuries can be avoided and patient satisfaction achieved.

In the present study, the highest number of maxillary ulcerations was seen in the posterior palatal seal area, which might be attributed to over-carving of the maxillary master cast in this area because some dentists have a misconception that over-carving of the maxillary master cast in this area results in greater retention of maxillary denture. The second highest frequency of maxillary mucosal injures was seen in the buccal slope of the residual ridge, which is attributed to bony undercuts and irregularities in the canine and molar areas of the maxilla. The denture-bearing mucosa in these areas is very thin and therefore susceptible to injury and ulceration.^1^

Kivoviks reported greater frequency of ulcerations among men compared to women.^2^ He believes that dietary factors play a role in the development of mucosal injuries during the first few days post-insertion and men usually consume hard foods and that is why they had more traumatic ulcerations compared to women.^2,8^ In the present study, no significant differences were observed in the number of ulcers between men and women. However, it is important to instruct patients how to correctly use their dentures and preferably consume soft foods during the first few days following denture placement.^1^

Considering the limitations of this study, it is once again emphasized that denture placement must not be the final patient-clinician encounter when treating complete denture patients and denture adjustments are very important clinical phases of denture fabrication and essential for patient care.

Since the highest number of ulcerations were detected in the borders, adequate extension of flanges during border molding and using pressure indicator methods before the delivery of dentures play an important role in preventing mucosal injuries and increasing patient comfort. In addition, patients should be trained how to correctly use their dentures. Consumption of soft foods should also be recommended.
